# Family Functioning in Families of Children with an Anxiety Disorder, with and without Autism Spectrum Disorder

**DOI:** 10.1007/s10803-025-06894-w

**Published:** 2025-06-03

**Authors:** Annelieke Hagen, Anke M. Klein, Susan M. Bögels, Bonny F. J. A. van Steensel

**Affiliations:** 1 Department of Developmental and Educational Psychology, Institute of Psychology, Leiden University, Wassenaarseweg 52, 2333 AK Leiden, The Netherlands; 2 Department of Developmental Psychology, University of Amsterdam, Amsterdam, The Netherlands; 3 Department of Child Development and Education, University of Amsterdam, Amsterdam, The Netherlands

**Keywords:** Family functioning, Anxiety disorder, Autism, Children, Adolescents, Cognitive behavioral therapy

## Abstract

This study compared family functioning among families of children with anxiety disorders, with and without autism spectrum disorder (ASD), and typically developing children. We evaluated (1) group differences in family functioning, (2) changes in family functioning following child-focused cognitive behavioral therapy for anxiety, and (3) whether pre-treatment family functioning predicted anxiety reduction. Participants were 264 children (aged 7–18 years) and their parents (251 mothers and 172 fathers). Groups consisted of children with anxiety disorders without ASD (*n* = 95), children with both conditions (*n* = 79), and typically developing children (*n* = 90). Children and parents completed the Family Functioning Scale, which assesses relational functioning and system maintenance. As expected, families of children with both anxiety disorders and ASD reported lower relational functioning and higher system maintenance than controls. System maintenance was also higher in these families than in the anxiety-only group (per both parents). Unexpectedly, the anxiety-only group did not differ from the control group. Additionally, cognitive behavioral therapy did not improve family functioning in either clinical group, nor did pre-treatment family functioning predict anxiety reductions. Families of children with both anxiety and ASD showed poorer family functioning than both other groups. Future research should examine whether these challenges stem from their co-occurrence or are specific to ASD.

Anxiety disorders are highly prevalent in children and are among the mental disorders with the earliest onset. They are associated with considerable impairment (Bandelow & Michaelis, 2015) and can lead to decreased participation in age-appropriate activities such as playdates, sports, and school. Additionally, anxiety is often characterized by challenges in peer relationships and may cause difficulties in peer acceptance and in forming and maintaining friendships (Kingery et al., 2010). The avoidance of age-appropriate activities and difficulties with peer acceptance may cause parents to feel helpless or frustrated, thereby influencing the functioning of the entire family. Fortunately, there is substantial evidence that anxiety disorders in children can be effectively treated with cognitive behavioral therapy (CBT; e.g., Bodden et al., 2008; Connolly et al., 2011; James et al., 2020; Perihan et al., 2020; Reynolds et al., 2012). However, much less is known about the family functioning in children with anxiety disorders and co-occurring conditions, such as children with autism spectrum disorder (ASD) and anxiety disorders, or about whether the family dynamics change after having followed CBT for anxiety disorders. Therefore the current study’s aim was to not only get more insight in family functioning in children with anxiety disorders, with and without ASD, but also to examine whether family functioning changed after these children have followed treatment for their anxiety disorders, and whether family functioning is related to anxiety symptom decreases after treatment.

Family functioning, which is referred to as the extent to which families engage in effective communication, handle daily routines, and foster positive interpersonal relationships, is a concept that has multiple dimensions (Zubrick et al., 2000). Often, two dimensions described in a factor analytic study of the family functioning scale are used to describe family functioning: family relationship and system maintenance (Bloom, 1985). A good family relationship is indicated by a more cohesive, expressive, outgoing, and supportive family environment. Higher system maintenance, which is often seen as less desirable, is characterized by a less organized, more hierarchical, and authoritarian family structure, with more enmeshment and a stronger external locus of control. Other definitions of family functioning are used in the literature as well; however, these terms are usually connected to and overlap with the dimensions put forward by Bloom (1985).

Poor family functioning characterized by lower family relational functioning and higher levels of system maintenance—is associated with increased psychological distress in parents, higher levels of behavioral problems, lower levels of prosocial behavior (Renzaho et al., 2013), *and* childhood anxiety. Jongerden et al. (2015), for example, found that families with a child diagnosed with an anxiety disorder reported higher levels of family conflict (i.e., controlling and constraining interactions) and lower levels of family relationship (i.e., cohesion and supportive interactions) compared to families of typically developing children. Several review studies have similarly concluded that families with a child diagnosed with anxiety disorders report lower levels of family functioning; however, it should be noted that there are also studies that did not find this relationship (for overviews, see Bögels & Brechman-Toussain, 2006; Drake & Ginsburg, 2012; Scully et al., 2020).

While anxiety impacts a child’s development and can influence family functioning, even greater challenges are faced by families with a child who has both anxiety disorders and other mental problems. Children with ASD frequently experience anxiety disorders (Bougeard et al., 2021; Lai et al., 2019; Micai et al., 2023; van Steensel et al., 2011). Higher levels of anxiety in children with ASD are associated with more interference in daily life, including avoidance of certain activities, behavioral problems, participation in home and community activities, and a lower self-reported quality of life (Adams & Emerson, 2021; Ambrose et al., 2022; Farrugia & Hudson, 2006; van Steensel et al., 2012). Higher levels of anxiety are also thought to have a negative impact on child functioning, over and above the impairments due to ASD symptoms (van Steensel et al., 2012). Conversely, children with anxiety disorders often display higher levels of ASD symptoms (Pine et al., 2008; Towbin et al., 2005; van Steensel et al., 2012), and there is behavioral overlap between anxiety and ASD symptoms, stimulating researchers to explore underlying mechanisms (e.g., sensory over-responsivity; Cummings et al., 2024) and mediators (e.g., executive functioning, emotion regulation and control; Fernandez-Prieto et al., 2021) to gain more insight in their relationship.

The impact of having a child with ASD in the family has been researched quite intensively (e.g., Davis & Carter, 2008; Myers et al., 2009; Pisula & Porębowicz-Dörsmann, 2017), but, to the authors’ knowledge, no research has specifically considered the presence of anxiety disorders in children with ASD. A recent systematic review of family functioning in families of children with ASD found that family functioning is generally more problematic for these families than for families of typically developing children (for a systematic review, see Desquenne Godfrey et al., 2024). However, this review also reported mixed findings regarding specific dimensions of family functioning, such as cohesion, organization, communication, conflict management, personal functioning within the family, and system control. Lower general family functioning in these families was shown to be associated with higher parental demand, including poorer quality of life, mental health, and emotional well-being of parents (Desquenne Godfrey et al., 2024). This review also showed that, while the severity of ASD is not associated with family functioning, certain comorbid problems—including behavioral problems, anxiety, and intellectual challenges—might be related to poorer family functioning. This underlines the importance of studying families with children who have anxiety disorders, both with and without ASD. Given the high prevalence of anxiety disorders in children with ASD, it is important to understand the differences and similarities between children with an anxiety disorder with or without ASD and to use this knowledge to provide guidelines for improving treatment outcomes.

Anxiety disorders in children, with and without ASD, can be effectively treated with cognitive behavioral therapy (e.g., Bodden et al., 2008; Connolly et al., 2011; James et al., 2020; Perihan et al., 2020; Reynolds et al., 2012). However, a significant minority of children who receive CBT for anxiety disorders continue to experience significant problems following treatment (for a review, see James et al., 2020). CBT treatment outcomes are typically measured only in terms of changes in the child’s diagnostic status or the severity of the disorder, rather than changes in parenting style or family functioning, despite the general belief that parental and family factors play an important role in the etiology and maintenance of childhood anxiety disorders (Breinholst et al., 2012; Drake & Ginsburg, 2012). A recent meta-analytic review shows that, although family components in CBT programs typically target at least one key parenting mechanism, many studies do not measure parent or family functioning at both pre- and post-treatment (Peris et al., 2021). The few studies that do examine family components have found that anxiety-focused CBT can improve family functioning, showing increases in family relational functioning, warmth and acceptance, and decreases in dysfunctional family control (Bögels & Siqueland, 2006; Crawford & Manassis, 2001; Jongerden & Bögels, 2015; Keeton et al., 2013; Siqueland et al., 2005). Additionally, some evidence suggests that less dysfunctional family functioning at baseline predicts better treatment outcomes (Crawford & Manassis, 2001; Ginsburg et al., 2018; Victor et al., 2007). However, the findings are inconsistent across studies, with variations in effect sizes and differences between parent and child reports. More importantly, these studies did *not* include children with ASD. The mixed findings in treatment studies, together with the evidence that family functioning is related to both ASD and to child anxiety, highlight the importance of better understanding how family factors relate to anxiety in children with and without ASD.

In the present study, we aimed to (1) compare the family functioning of families with children who have an anxiety disorder, both with or without ASD, and a control group of typically developing children without a DSM classification; (2) examine whether family functioning in both clinical groups changed after child-focused CBT for anxiety disorders and whether these changes differed between children with and without ASD; and finally (3) explore whether the quality of family functioning (measured at pre-treatment) predicted a greater improvement in anxiety symptoms after treatment. We used the Family Functioning Scale (FFS; Bloom, 1985) to assess family functioning across two dimensions: family relational functioning and system maintenance. We hypothesized that family relational functioning would be lower and system maintenance would be higher in families of children with both an anxiety disorder and ASD compared to both other groups. Additionally, we expected that children with only an anxiety disorder would also show lower family functioning than the control group of typically developing children. Finally, we hypothesized that family functioning would improve after CBT for both clinical groups and that better pre-treatment family functioning (i.e., higher family relational functioning and lower system maintenance) would predict a greater reduction in anxiety symptoms post-treatment.

## Methods

### Participants

Participants were 264 children aged 7–18 (57% boys, mean age 12.19 years, 96% caucasian), and their parents (251 mothers and 172 fathers participated), categorized into three groups: children diagnosed with an anxiety disorder without ASD (*n* = 95; hereafter referred to as the AD group), children diagnosed with ASD and an anxiety disorder (*n* = 79; hereafter referred to as the ASD + AD group), and typically developing children from the general population (*n* = 90; hereafter referred to as the control group); see Table 1 for the baseline characteristics of the three groups (Table 1).

**Table 1:** Baseline characteristics of participants

Baseline characteristic	Total sample (*N* = 264)		ASD + AD group (n = 79)		AD group (n = 95)		Control group (n = 90)	
Child								
Age *M* (*SD*)	12.19	(2.93)	11.76	(2.68)	12.85	(2.81)	11.88	(3.15)
Gender	151♂	(57%)	58♂	(73%)	46♂	(48%)	47♂	(52%)
	113♀	(43%	21♀	(27%)	49♀	(52%)	43♀	(48%)
Having a sibling (yes)	240	(91%)	67	(85%)	83	(87%)	90	(100%)
Comorbid disorder present (yes) based on ADIS-CP								
Obsessive compulsive disorder	28	(11%)	12	(15%)	16	(17%)	0	(0%)
Post-traumatic stress disorder	16	(6%)	6	(8%)	10	(11%)	0	(0%)
Mood disorder (depression/dysthymia)	42	(16%)	14	(18%)	28	(29%)	0	(0%)
Attention deficit hyperactivity disorder	15	(6%)	10	(13%)	5	(5%)	0	(0%)
Oppositional defiant disorder	5	(2%)	2	(3%)	3	(3%)	0	(0%)
Education								
Special education	20	(8%)	18	(23%)	2	(2%)	0	(0%)
Primary education	119	(45%)	33	(42%)	35	(37%)	51	(57%)
Secondary education	123	(47%)	28	(35%)	57	(60%)	38	(42%)
Lowest level	28	(23%)	8	(29%)	16	(28%)	4	(11%)
Middle level	37	(30%)	9	(32%)	20	(35%)	8	(21%)
Highest level	58	(47%)	11	(39%)	21	(37%)	26	(68%)
Other	2	(< 1%)	0	(0%)	1	(1%)	1	(1%)
Mother *n* (%)	251	(95%)	78	(99%)	90	(95%)	83	(92%)
Age *M* (*SD*)	42.61	(4.96)	42.71	(4.66)	42.74	(4.67)	42.37	(5.57)
Education *M* (*SD*)	5.76	(1.94)	5.56	(1.88)	5.41	(1.84)	6.34	(2.01)
Father *n* (%)	172	(65%)	57	(72%)	70	(74%)	45	(50%)
Age *M* (*SD*)	45.11	(5.17)	44.75	(4.93)	45.11	(5.06)	45.56	(5.65)
Education *M* (*SD*)	6.27	(1.97)	6.32	(1.83)	5.87	(2.15)	6.84	(1.71)

Maternal and paternal education range from 1 (no education) to 9 (university)

Inclusion criteria for the two clinical groups were: (a) the presence of at least one anxiety disorder, and (b) the willingness of at least one caregiver and the child to participate in the study. Exclusion criteria were: (a) IQ below 70, (b) untreated psychotic disorder, (c) acute risk for suicide, and (d) current physical or sexual abuse. Additionally, any medication had to be stable in dosage one month prior to and during the study and no other treatment was received concurrently with the in-study CBT treatment (of note, 25 children of the ASD + AD group used medication; nine received psychostimulants, eight antipsychotics, three anxiolytics, four psychostimulants and antipsychotics, and one child received antipsychotic and anxiolytic; and eight children of the AD group used medication; one child received psychostimulants, two antipsychotics, and five anxiolytics). The inclusion criteria for the control group were: (a) the absence of a DSM-IV disorder (previously diagnosed by a clinician), and (b) currently not consulting or being referred to a mental health care specialist. In the control group, 11 children (12.2%) met the criteria for an anxiety disorder according to the ADIS-C/P assessment. These children were not seeking treatment for their anxiety and had not been referred to mental health care. They were not excluded from the study due to the high prevalence of anxiety disorders in the general population; their exclusion might have resulted in an overly healthy control group.

The current study is a secondary analysis of previously collected data, that is, the children in the two clinical groups were enrolled in a study comparing treatment effectiveness of CBT for anxiety in children with an anxiety disorder with and without ASD (see van Steensel & Bögels, 2015, for a detailed description of participants and procedure). Medical ethical approval for the study was obtained. The control group was recruited separately using convenience sampling. Ethical approval for the control group was obtained through the University of Amsterdam.

## Materials and Intervention

### Intervention

Children in the AD and ASD + AD groups received CBT following the “Discussing + Doing = Daring” protocol, developed to treat anxiety disorders in clinically referred children (Bögels et al., 2008). This intervention is widely used in the Netherlands and Belgium and is considered a standard child-focused CBT protocol for treating anxiety disorders. The treatment protocol has been found to be highly effective in a large Dutch sample of referred children with anxiety disorders without ASD (Bodden et al., 2008). The effectiveness study from which the current clinical sample was taken showed that this CBT program was also effective in children with ASD with anxiety disorders and, on most outcome measures, the program was not more or less effective for children with ASD and anxiety than for children with anxiety disorders without ASD (van Steensel & Bögels, 2015). There were no a priori adaptations made to the intervention protocol for the children with ASD. In addition, via audiotaped treatment sessions, independent coders assessed treatment integrity by rating the session treatment goals (2 to 6 goals per session) as 0 (not accomplished), 1 (somewhat accomplished) or 2 (totally accomplished). The mean goal rating scores were not different for the ASD + AD group and the AD group (mean scores of 1.43 and 1.39, respectively).

### Family Functioning

Family functioning was assessed with the FFS (Bloom, 1985). The FFS is a factor-analytic combined version of four family questionnaires: the Family Environment Scale, the Family Concept Q Sort, the Family Adaptability and Cohesion Evaluation Scales and the Family Assessment Measure. The FFS is a questionnaire with 60 items that are rated on a 4-point scale (from 1 = very untrue to 4 = very true), and was filled out by the child, mother, and father. Two dimensions can be distinguished: family relational functioning and system maintenance, also referred to as the family control dimension (Jongerden & Bögels, 2015). A higher score on the family relational functioning dimension indicates a more cohesive, expressive, outgoing, and supportive family. An example item is: “family members really help and support one another.” A higher score on the system maintenance dimension indicates a less organized, more hierarchical, and authoritarian family with more enmeshment and a higher external locus of control. An example item is: “there is strict punishment for breaking rules in our family.” Internal consistency of the family relational functioning dimension in the current study was good for child, mother, and father ratings (α coefficients ranging from 0.87 to 0.89). Internal consistency of the maintenance dimension was questionable to acceptable for child, mother, and father ratings (α coefficients ranging between 0.64 and 0.75).

### Anxiety Diagnosis

Anxiety disorders were assessed with the Anxiety Disorders Interview Schedule for DSM-IV: Child and Parent Versions (ADIS-IV-C/P; Silverman & Albano, 1996). The ADIS-C/P is a semi-structured interview designed to diagnose anxiety and related disorders in children and adolescents. In addition to providing diagnoses based on parent report (ADIS-P) and child report (ADIS-C), the interview provides a combined diagnosis, which was used in this study. As part of the interview, the severity of the anxiety disorder was rated on a 0–8 scale. The severity rating is based on the level of interference and avoidance, the intensity of anxiety, the number of symptoms, and the impression of the administrator. A rating of 4 or higher indicates clinical significance, meeting the criteria for an anxiety disorder diagnosis. The ADIS-IV demonstrates good test–retest reliability (Silverman et al., 2001) and concurrent validity (Wood et al., 2002), and has been used in studies assessing anxiety in children with ASD (e.g., Reaven et al., 2012; Wood et al., 2009). Interrater reliability was not assessed for the current sample. However, in previous studies by our research group, interrater reliability for the ADIS-C/P has been found to be high, with kappa ranging between 0.73 and 1.00. For further details, see van Steensel and Bögels (2015).

### Anxiety Symptoms

Children and parents rated the child’s anxiety symptoms by each completing the Screen for Child Anxiety Related Emotional Disorders-71 (SCARED-71; Bodden et al., 2009). The SCARED-71 contains 71 items that are rated on a 3-point scale (0 = almost never, 1 = sometimes, 2 = often). An example item is: “I am afraid of heights.” The psychometric properties of the SCARED-71 have been found to be robust in both typically developing and ASD samples (Bodden et al., 2009; van Steensel et al., 2013). Internal consistency in the current sample was excellent, with Cronbach’s alpha across assessments and groups ranging from 0.91 to 0.96.

### Procedure

Participants in the two clinical groups were children who were referred to one of seven participating community mental health care centers in The Netherlands for the treatment of an anxiety disorder. After the center's regular intake and assessment procedure, DSM-IV-TR diagnoses were established by a multidisciplinary team based on clinical evaluations, including interviews with the parent(s) and child, observations of child-parent interactions and/or school observations, diagnostic assessments, and psychiatric consultations. In addition, the Autism Diagnostic Interview-Revised (ADI-R; Lord, et al., 1994) was administered to the parents of 60 children in the ASD + AD group. The interview was administered by an independent researcher, and the scores on the ADI-R were not shared with the clinical staff who were responsible for the ASD diagnosis. Mean scores on the ADI-R were respectively 16.33, 11.17 and 4.17 for the social, communicative and repetitive domains; and 97%, 88% and 70% met the thresholds for these domains. IQ was estimated based on school performance. That is, children in the Netherlands complete an annual school assessment to evaluate their academic skills. The scores on the assessment provide information about how well the child performs academically compared to other children (with scores presented in the far below, below, average, above average or far above average range). The (final) score in primary school provides an indication of which secondary school level (low, middle or high) is most appropriate for a child, or whether, for example, attending a practical education program (typically attended by children with an IQ below 80) is more suitable.

If a child met all inclusion criteria and the family provided informed consent, the child was included in the study and assigned to the AD or ASD + AD group based on their diagnosis. Following the first assessment, children received CBT for their anxiety disorder. Assessments were conducted before the start of the treatment (pretest), directly following treatment (posttest), at 3 months after treatment (follow-up 1), 1 year after treatment (follow-up 2), and 2 years after treatment (follow-up 3). The control group was recruited through advertisements in schools, day-care facilities, and convenience sampling by University of Amsterdam graduate students and was assessed only once.

### Analyses

To examine group differences in family functioning prior to treatment, MANCOVAs were conducted with gender and age as covariates (research question 1). Analyses were conducted separately for the family relational functioning and the system maintenance dimensions of the FFS, with separate analyses for child, mother, and father reports. Assumptions of normality were violated for the family relational functioning and system maintenance subscales based on child reports, as well as for the family relational functioning subscale based on mother reports. However, Q-Q plot inspections indicated only minor deviations from normality. Given the large sample size and the minor deviations, these violations were deemed inconsequential for the analyses. Five outliers were identified but removing them did not alter the results. We therefore retained the entire sample for analyses. Additionally, a violation of the homogeneity assumption was observed for the family relational functioning subscale based on child reports; however, bootstrapping with 5000 samples yielded identical results, indicating that the model remained robust despite this violation.

To examine changes in family functioning following treatment, we used multilevel analyses (research question 2). All models employed a first-order autoregression (AR1) covariate structure which has been shown to be suitable for longitudinal data (Bollen & Curran, 2004, 2006; Curran & Bollen, 2001). Scores on the family relational functioning dimension and system maintenance dimension were dependent variables. Time (pre-treatment, 3-month follow-up, 1-year follow-up, and 2-year follow-up, each contrasted against post-treatment), diagnosis (presence or absence of ASD), and their interaction (time × diagnosis) were entered as predictors, with age and gender included as covariates. Analyses were conducted separately for child, mother, and father reports.

To explore whether family functioning at pre-treatment predicted a reduction in anxiety symptoms after treatment, we again used multilevel analyses (research question 3). Total anxiety scores on the SCARED-71 served as the dependent variable. Time (pre-treatment, 3-month follow-up, 1-year follow-up and 2-year follow-up), diagnosis (presence or absence of ASD), FFS score at pre-treatment (family relational functioning/system maintenance), and their interaction (FFS × diagnosis and FFS × time) were entered as predictors, with age and gender included as covariates. Analyses were conducted separately for child, mother, and father reports, using both the SCARED-71 and FFS completed by the respective informants (e.g., for models based on child report, the child-completed FFS and SCARED-71 were used).

Since all analyses were conducted separately for child, mother, and father reports as well as for the two dimensions of the FFS, a Bonferroni correction for six models was applied to each research question. Thus, a *p-*value of less than 0.0083 (calculated as 0.05/6) was considered significant.

## Results

### Descriptives

The groups significantly differed from each other in terms of age, *F*(2, 261) = 3.89 (*p* = 0.022), and gender, *Χ*^*2*^(2) = 12.39 (*p* = 0.002). Children in the AD group were significantly older than those in the ASD + AD group, though neither clinical group differed significantly in age from the control group. Additionally, the ASD + AD group had significantly more boys than both the AD and control groups (see Table 1). Therefore, we controlled for age and gender in all analyses.

### Family Functioning at Pre-Treatment

To compare family functioning (family relational functioning and system maintenance) between the three groups (ASD + AD group, AD group, and control group), several MANCOVAs were conducted (see Table 2). As expected, family relational functioning was significantly lower for the ASD + AD group compared to the control group (based on father, mother, and child reports). Unexpectedly, no significant differences were found between the AD group and the control group, or between the ASD + AD and the AD group.

**Table 2:** Means and standard deviations of pre-treatment family functioning (Relationship and system maintenance dimensions) in children with AD and ASD, AD only, and healthy controls

Variable	ASD + AD group		AD group		Control group		*F, p*	Contrast
Child report *N*	65		89		90			
Relationship	88.52^b^	(11.99)	92.83^a^	(14.25)	96.72^a^	(10.22)	*F*(2, 239) = 7.48, *p* < .001^**^	Control vs AD, *p* = .114 Control vs AD + ASD, *p* < .001** AD vs AD + ASD, *p* = .018
System maintenance *M* (*SD*)	62.08^b^	(7.14)	58.52^a^	(8.38)	57.77^a^	(7.90)	*F*(2, 239) = 5.86, *p* = .003^*^	Control vs AD, *p* = .291 Control vs AD + ASD, *p* < .001** AD vs AD + ASD, *p* = .018
Mother report *N*	72		87		82			
Relationship *M (SD*)	91.38^b^	(11.29)	94.20^a^	^b^ (10.59)	98.05^a^	(10.59)	*F*(2, 236) = 6.17, *p* = .002^*^	Control vs AD, *p* = .054 Control vs AD + ASD, *p* < .001** AD vs AD + ASD, *p* = .102
System maintenance *M* (*SD*)	59.58^b^	(7.80)	55.82^a^	(6.89)	54.91^a^	(7.25)	*F*(2, 236) = 6.94, *p* = .001^*^	Control vs AD, *p* = .360 Control vs AD + ASD, *p* < .001** AD vs AD + ASD, *p* = .007^*^
Father report *N*	55		66		45			
Relationship *M* (*SD*)	89.15^c^	(9.65)	93.36^b^	(10.80)	98.27^a^	(8.36)	*F*(2, 161) = 9.69, *p* < .001^**^	Control vs AD, *p* = .020 Control vs AD + ASD, *p* < .001** AD vs AD + ASD, *p* = .019
System maintenance *M* (*SD*)	61.31^b^	(6.90)	57.36^ab^	(7.13)	54.78^a^	(6.28)	*F*(2, 161) = 11.21,* p* < .001^**^	Control vs AD, *p* = .070 Control vs AD + ASD, *p* < .001** AD vs AD + ASD, *p* = .002^*^

The covariate child age was significant for the Relationship dimension based on child and mother report, and for the System maintenance dimension based on child report. With younger children scoring higher on both the relationship and system maintenance dimensions. Superscript letters are used to indicate which groups differ significantly from each other; groups with the same letter do not differ significantly

^*^p < .0083 (Bonferroni corrected for multiple comparisons: .05/6), ^**^p < *.*001

In line with our hypothesis, system maintenance was significantly higher for the ASD + AD group compared to the control group (based on father, mother, and child reports), and for the ASD + AD group compared to the AD group (based on father and mother reports). Unexpectedly, no significant differences in system maintenance were found between the AD group and the control group.

### Changes in Family Functioning Following Treatment

To examine changes in family functioning following treatment in the AD and ASD + AD groups, we conducted multilevel analyses (see Tables 3 and 4; see Appendix A for means and standard deviations). Unexpectedly, no significant effects of time were observed for family relational functioning, indicating that it did not significantly change following treatment. We did find a significant main effect of age across all time points: higher child age was associated with significantly lower relational functioning (based on child, mother, and father reports). Additionally, for the system maintenance dimension, we did not find the expected significant effects of time, indicating that system maintenance did not significantly change over time.

**Table 3:** Multilevel analyses of family functioning on the relationship dimension over time (Intervention took place between pre-test and post-test) for child, mother, and father report with post-test as reference category

	Child			Mother			Father		
Parameter	Estimate	SE	*p*	Estimate	SE	*p*	Estimate	SE	*p*
Intercept	− .10	.220	.639	− 0.19	.226	.413	− 0.20	.256	.449
ASD (0 = no ASD, 1 = ASD)	− 0.39	.162	.018	− 0.39	.161	.017	− 0.47	.188	.014
Pre-treatment	− 0.13	.080	.104	− 0.15	.076	.055	− 0.16	.091	.088
3-month FU	− 0.01	.084	.865	− 0.10	.080	.214	− 0.05	.101	.609
1-year FU	− 0.04	.116	.728	0.05	.113	.653	0.13	.141	.376
2-year FU	− 0.03	.132	.843	0.04	.129	.770	− 0.11	.162	.498
Child gender (0 = boy, 1 = girl)	0.22	.129	.086	0.28	.133	.037	0.32	.153	.041
Child age	− 0.32	.064	< .001^**^	− 0.27	.065	< .001^**^	− 0.20	.074	.008^*^
ASD * pre-test	0.04	.123	.758	0.11	.112	.320	0.07	.140	.614
ASD * 3-month FU	0.08	.130	.525	0.02	.117	.879	0.02	.154	.899
ASD * 1-year FU	0.17	.174	.328	− 0.10	.163	.522	− 0.26	.213	.231
ASD * 2-year FU	− 0.02	.197	.909	− 0.06	.185	.752	0.12	.245	.614

^*^p < .0083 (Bonferroni corrected for multiple comparisons: .05/6), ^**^p < *.*001

**Table 4:** Multilevel analyses of family functioning on the system maintenance dimension over time (Intervention took place between pre-test and post-test) for child, mother, and father report with post-test as reference category

	Child			Mother			Father		
Parameter	Estimate	SE	*p*	Estimate	SE	*p*	Estimate	SE	*p*
Intercept	0.23	.216	.280	0.40	.225	.075	0.09	.247	.727
ASD (0 = no ASD, 1 = ASD)	0.21	.168	.211	0.30	.164	.070	0.44	.190	.023
Pre-test	0.08	.097	.391	0.07	.085	.432	0.08	.104	.428
3-month FU	− 0.07	.102	.473	0.01	.090	.952	0.01	.115	.949
1-year FU	− 0.20	.137	.151	− 0.24	.124	.055	− 0.24	.158	.136
2-year FU	− 0.36	.151	.017	− 0.26	.142	.064	− 0.06	.177	.716
Gender (0 = boy, 1 = girl)	− 0.20	.124	.103	− 0.35	.131	.009	− 0.20	.146	.183
Age	− 0.06	.061	.350	0.06	.065	.340	0.10	.071	.179
ASD * pre-test	0.09	.148	.525	0.15	.126	.226	0.08	.160	.600
ASD * 3-month FU	0.06	.157	.724	0.05	.132	.720	0.02	.176	.898
ASD * 1-year FU	0.08	.205	.687	0.04	.178	.839	0.39	.238	.105
ASD * 2-year FU	0.26	.225	.256	0.08	.204	.708	− 0.06	.268	.831

^*^p < .0083 (Bonferroni corrected for multiple comparisons: .05/6), ^**^p < *.*001

### Family Functioning as a Predictor of Treatment Outcome

To examine whether family functioning predicted treatment outcome for the AD and ASD + AD groups, we conducted multilevel analyses (see Tables 5 and 6). Significant main effects of time were observed on anxiety symptoms (SCARED), indicating a decrease in anxiety symptoms over time. These effects were consistent across all models. Mother and father reports also showed a main effect of diagnosis, with the ASD + AD group exhibiting significantly higher anxiety levels across assessments. Additionally, child reports indicated a main effect of gender, with girls reporting higher anxiety scores across time points than boys.

**Table 5:** Multilevel analyses of child anxiety symptoms over time for child, mother and father report, with pre-treatment family functioning on the relationship dimension as a predictor

	Child			Mother			Father		
Parameter	Estimate	SE	*p*	Estimate	SE	*p*	Estimate	SE	*p*
Intercept	− 0.14	.206	.945	.093	.208	.655	.268	.240	.267
ASD (0 = no ASD, 1 = ASD)	.333	.126	.009	.628	.125	< .001^**^	.551	.147	< .001^**^
Post-test	− .623	.065	< .001^**^	− .628	.054	< .001^**^	− .907	.063	< .001^**^
3-month FU	− .769	.085	< .001^**^	− .830	.072	< .001^**^	− .820	.087	< .001^**^
1-year FU	− .891	.099	< .001^**^	− .849	.086	< .001^**^	− .844	.106	< .001^**^
2-year FU	− 1.120	.107	< .001^**^	− 1.018	.093	< .001^**^	− 1.028	.117	< .001^**^
Relationship	− .000	.090	.996	− .184	.096	.056	− .184	.105	.083
Gender (0 = boy, 1 = girl)	.351	.123	.005^*^	.176	.125	.162	.105	.147	.474
Age	.052	.066	428	− .016	.064	.803	− .078	.073	.289
ASD * Relationship	− .070	.125	.577	.175	.121	.148	− .055	.148	.713
Post-test * Relationship	.003	.068	.961	− .045	.053	.392	.053	.064	.405
3-month * Relationship	− .026	.085	.759	− .012	.072	.863	.008	.088	.930
1-year FU * Relationship	− .026	.101	.795	.016	.085	.850	− .023	.107	.831
2-year FU * Relationship	− .079	.110	.474	− .008	.091	.931	.016	.116	.893

^*^p < .0083 (Bonferroni corrected for multiple comparisons: .05/6), ^**^p < *.*001

**Table 6:** Multilevel analyses of child anxiety symptoms over time for child, mother, and father report, with pre-treatment family functioning on the system maintenance dimension as a predictor

	Child			Mother			Father		
Parameter	Estimate	SE	*p*	Estimate	SE	*p*	Estimate	SE	*p*
Intercept	.010	.207	.963	.094	.201	.640	.305	.229	.186
ASD (0 = no ASD, 1 = ASD)	.315	.126	.014	.566	.122	< .001^**^	.477	.142	.001^*^
Post-test	− .620	.065	< .001^**^	− .627	.053	< .001^**^	− .913	.063	< .001^**^
3-month FU	− .763	.084	< .001^**^	− .830	.072	< .001^**^	− .816	.086	< .001^**^
1-year FU	− .885	.099	< .001^**^	− .854	.085	< .001^**^	− .847	.104	< .001^**^
2-year FU	− 1.115	.107	< .001^**^	− 1.023	.092	< .001^**^	− 1.033	.114	< .001^**^
System maintenance	.146	.087	.094	.297	.092	.001^*^	.250	.102	.016
Gender (0 = boy1 = girl)	.336	.124	.007^*^	.203	.121	.094	.098	.139	.485
Age	.074	.063	.241	.017	.059	.777	− .074	.068	.281
ASD*system maintenance	.068	.125	.584	− .179	.116	.125	.105	.140	.454
Post-test * system maintenance	− .173	.064	.008^*^	.047	.052	.374	− .096	.063	.128
3-month FU * system maintenance	− .205	.082	.013	.014	.070	.843	.070	.089	.427
1-year FU * system maintenance	− .193	.096	.045	.073	.083	.377	.054	.108	.615
2-year FU * system maintenance	− .152	.103	.141	.020	.089	.821	.031	.117	.790

^*^p < .0083 (Bonferroni corrected for multiple comparisons: .05/6), ^**^p < *.*001

Contrary to our expectations, no significant interaction effects were found for the models that included family relational functioning as a predictor (see Table 5). This indicates that family relational functioning at pre-treatment was not significantly associated with reductions in anxiety symptoms after treatment for either clinical group (based on child, mother, and father reports).

When we included system maintenance as a predictor, we also did not find the expected significant interaction effects (see Table 6). This suggests that lower levels of system maintenance at pre-treatment were not associated with greater reductions in anxiety symptoms following treatment. The only exception was a significant negative interaction effect between time and system maintenance based on child reports, but this effect was observed only post-treatment: a higher level of system maintenance was associated with a stronger decrease in anxiety symptoms immediately post treatment, but this effect did not persist in follow-up assessments. Additionally, a significant positive main effect of system maintenance based on mother reports indicated that higher levels of system maintenance at pre-treatment were associated with higher anxiety levels across time points.

## Discussion

The present study aimed to further clarify the role of family functioning in families of children with an anxiety disorder, with and without ASD. In addition, we tested whether family functioning would change after child-focused CBT for anxiety disorders and whether pre-treatment family functioning would predict treatment outcome. The results can be summarized as follows: (1) As expected, children with both AD and ASD showed lower family relational functioning and higher levels of system maintenance compared to controls (reported by children and parents) *and* to children with AD only (reported by parents). Contrary to our expectations, there were no significant differences between the children with AD and controls on family functioning; (2) Unexpectedly, family functioning did not significantly change following child anxiety CBT for both clinical groups and (3) relational functioning and system maintenance levels at pre-treatment did not predict anxiety symptom decrease.

The expected lower family relational functioning and higher levels of system maintenance for children with an anxiety disorder and ASD align with several previous studies (for a systematic review, see Desquenne Godfrey et al., 2024). This recent review concluded that general family functioning in families of children with ASD was lower than in typically developing children. Our study also showed that for children with both anxiety and ASD, system maintenance was significantly higher according to parents than in children with anxiety disorders alone. This finding could reflect less functional family dynamics in families of children with ASD, as system maintenance may be less adaptive in these families. For example, parents may exhibit similar ASD traits, which can influence family functioning (see Desquenne Godfrey et al., 2024, for further explanations). Alternatively, this result might indicate that children with ASD benefit from a different family dynamic characterized by clear expectations, stricter rules, routines, and limits. Setting clear boundaries and creating structured environments with well-defined expectations has been shown to be beneficial for children with ASD and their families (e.g., Osborne et al., 2008). Teaching parents how to implement these strategies is often a key component of ASD interventions (e.g., Schopler et al., 1995). This may explain why parents of children with both ASD and anxiety report higher levels of system maintenance—a dimension that includes items on hierarchy and rules—than parents of children with anxiety alone, raising questions about whether this is necessarily a negative outcome.

Differences in family relational functioning and system maintenance between children with an anxiety disorder and the control group align with previous studies (see Jongerden et al., 2015), but the differences between the groups were not statistically significant in the current study, which was unexpected. Interestingly, the family functioning of children with only anxiety disorders appears to be more similar to that of typically developing children than to children with both ASD and anxiety disorders. The reasons for the non-significant differences in family functioning between typically developing children and those with anxiety disorders alone remain unclear. It is possible that the difference in family functioning between these groups is relatively small and thus difficult to detect with the sample size in our study. Notably, the mean differences in anxiety levels between the control and anxiety groups were comparable to those observed by Jongerden et al. (2015). This suggests that the discrepancies with previous research may be less pronounced than initially assumed.

With regard to the effect of family functioning on treatment outcome, we did not find the expected improvements in family functioning following anxiety treatment for either clinical group, contrary to other studies (e.g., Bögels & Siqueland, 2006; Crawford & Manassis, 2001; Jongerden & Bögels, 2015; Keeton et al., 2013; Siqueland et al., 2005). For the children in the anxiety-only group, this result might be explained by the fact that, in our study, family functioning at pre-treatment was similar between these children and the control group. This similarity makes it challenging to determine whether family functioning could—or should—improve following child-focused CBT in this group. It also raises the question of whether there is indeed room for improvement in family functioning of children with an anxiety disorder (without ASD), and, more importantly, if such improvement is necessary, at least within the context of this study. For children with an anxiety disorder and ASD, however, we found that family functioning was significantly worse compared to controls and this remained stable across different time points. This indicates that child-focused CBT targeting anxiety did not change family functioning in children with both anxiety and ASD. It also suggests that if the target is to improve family functioning, additional help beyond anxiety-focused child-CBT is necessary.

Our third research aim focused on the predictive value of family functioning on treatment outcome. We expected that both higher family relational functioning and lower system maintenance at pre-treatment would predict a greater reduction in anxiety symptoms following treatment. However, no significant relationships were found for either clinical group, except for one interaction effect for system maintenance, as reported by children directly post-treatment. Since this effect did not persist at later follow-ups and was reported only by children (not parents), it may represent a minor, short-term change related to adjustments at home during therapy (e.g., the implementation and enforcement of therapy homework within the family) rather than a stable change in family dynamics. For the children with anxiety disorders only, this outcome might partly reflect a floor effect, their family functioning scores were comparable to those of the control group. For children with both anxiety and ASD, the results may be due to the treatment’s primary focus on anxiety rather than ASD. Since ASD is associated with lower family functioning, a treatment focused solely on anxiety may not change family functioning in this group. Alternatively, or additionally, it is important to note that on a group-level no family functioning changes were observed, and on a group-level pre-treatment family functioning was not found to be associated with symptom change. On an individual family level, or for some families, this may be different. For example, it could be that for some families the CBT program was helpful in improving their family functioning, and it may therefore be worth the effort for future studies to take a more within-group approach and examine for which—or for each—family if and how family functioning is related to anxiety, ASD, and treatment outcomes. Meanwhile, it remains unclear whether and how optimal family functioning and effective parental behaviors for children with ASD differ from the needs of typically developing children, underscoring the importance of considering the unique needs of (individual) families with ASD in future studies.

Strengths of the present study include the use of reports from children, mothers, and fathers to measure family functioning and anxiety, as well as the comparison between children with both anxiety and ASD, children with only anxiety disorders, and typically developing children. Additionally, the study featured long-term assessments up to two years after treatment. Despite these strengths, the study also had several limitations. First, the reliance on self-report measures may introduce bias and complicate interpretation. In the field of parenting behaviors and styles, clear evidence exists for discrepancies between self-report and observational measures (e.g., Morsbach & Prinz, 2006). Moreover, these discrepancies appear to be larger among parents with higher levels of distress or a lower socioeconomic status (Herbers et al., 2017). This is particularly relevant to the present study’s findings, as higher parental stress levels are often observed in parents of children with ASD but not in parents of children with an anxiety disorder (e.g., Hayes & Watson, 2013; Telman et al., 2017). Thus, including observational measures or behavioral family tasks could provide different insights and would be an important next step for a more in-depth analysis of the relationship between family functioning in children’s anxiety (both with and without ASD).

Second, the lack of cut-off scores for the FFS leaves it unclear to what extent the family functioning scores found in this study indicate concerning levels or merely reflect mild differences that are statistically significant but clinically irrelevant. Establishing clear cut-offs for problematic family functioning is complicated by the fact that different children and families may benefit from different family dynamics. Furthermore, it is not clear whether higher system maintenance is always detrimental; rather, it may serve as a family dynamic that is beneficial in certain situations. For example, it may be that stricter rules and control can be helpful when learning new tools and practicing with overcoming anxiety (e.g., doing exposure exercises at home). Future studies could therefore focus on parental and familial adaptability to the needs of family members, specific situations, and family satisfaction, rather than solely on family functioning itself. To advance research on family factors and functioning, it is important to better understand what constitutes problematic family functioning and how to measure it consistently and reliably.

Lastly, as our study did not include a group of families with children who only had ASD, we cannot ascertain whether the lower family functioning in the families with children with both anxiety and ASD resulted from the combination of both conditions, or was solely due to ASD. In addition, there are many other factors to consider in relation to family functioning (e.g., socioeconomic status, parental stress, other family members with diagnoses or illnesses; see Desquenne Godfrey et al., 2024), and how these factors are to be considered—as predictors, moderators, mediators, or simply associated variables—in relation to family functioning and (mental) health remains unclear (see, for example, Booysen et al., 2021; Masarik & Conger, 2017; Pedersen & Revenson, 2005). These factors were not included in the current study, and therefore their possible effect on family functioning could not be examined, nor controlled for, which is a limitation of this study and requires further investigation.

In conclusion, the results of this study indicated lower family functioning, particularly in families of children with both an anxiety disorder *and* ASD, and not in families with children with only an anxiety disorder. Notably, parents of children with both anxiety and ASD reported higher levels of system maintenance than parents of children with anxiety disorders alone. Future studies are needed to determine whether the lower family functioning observed in families of children with both anxiety and ASD is attributable to the combination of the two disorders or solely to ASD. Additionally, our findings revealed that child-focused CBT did not result in changes in family functioning and that family functioning was not predictive of the decrease in anxiety levels. This may suggest that family functioning is not a critical factor in treating childhood anxiety. We may also need to reconsider how we define and measure family functioning; thereby taking into account the lack of clinical cut-of scores, the reliance on self-report, and the possibility of measuring other constructs of the family (e.g., families’ adaptability, satisfaction with the family’s functioning). While these results do not provide definitive conclusions regarding the role of family functioning in children with anxiety disorders, with and without ASD, they provide a good starting point for gaining more insight into this area.

## Appendix A

See Table

**Table 7:** Means and standard deviations of family functioning across timepoints on the relationship and system maintenance dimensions for the autism plus anxiety disorder group, the anxiety disorder only group, and control group

Variable	Pre-assessment			Post-assessment		3-month FU			One-year FU		Two-year FU	
ASD + AD group												
Child report *N*	65			53		49			43		45	
Relationship *M* (*SD*)	88.52		(11.99)	89.91	(14.17)	90.73	(15.87)		92.63	(12.22)	89.38	(14.91)
System maintenance *M* (*SD*)	62.08		(7.14)	60.02	(8.71)	60.43	(8.62)		59.09	(7.24)	59.73	(9.61)
Mother report *N*	72			59		54			45		49	
Relationship *M* (*SD*)	91.38		(11.29)	91.92	(12.45)	90.89	(11.55)		91.27	(12.75)	91.16	(13.78)
System maintenance *M* (*SD*)	59.58		(7.80)	57.93	(7.27)	58.04	(7.68)		57.82	(8.18)	56.90	(8.08)
Father report *N*	55			38		34			26		26	
Relationship *M* (*SD*)	89.15	(9.65)		89.42	(10.31)	89.56		(9.10)	88.04	(9.13)	90.27	(8.58)
System maintenance *M* (*SD*)	61.31	(6.90)		59.76	(7.15)	60.18		(7.24)	61.27	(6.42)	58.46	(7.61)
AD group												
Child report *N*	89			72		66			49		52	
Relationship *M* (*SD*)	92.83	(14.25)		94.76	(12.34)	94.58		(13.65)	93.33	(13.93)	94.62	(11.68)
System maintenance *M* (*SD*)	58.52	(8.38)		58.24	(9.20)	57.86		(8.66)	57.02	(10.16)	54.75	(9.12)
Mother report *N*	87			70		62			44		49	
Relationship *M* (*SD*)	94.20	(10.59)		96.04	(9.87)	95.18		(10.05)	97.09	(10.09)	96.94	(11.81)
System maintenance *M* (*SD*)	55.82	(6.89)		55.29	(6.82)	55.13		(6.23)	54.00	(7.10)	52.90	(6.48)
Father report *N*	66			56		43			31		32	
Relationship *M* (*SD*)	93.36	(10.80)		94.87	(9.84)	94.67		(9.84)	97.45	(11.05)	94.13	(10.52)
System maintenance *M* (*SD*)	57.36	(7.13)		56.66	(7.35)	56.77		(6.17)	55.06	(7.81)	56.34	(6.29)
Control group												
Child report *N*	90			NA		NA			NA		NA	
Relationship *M* (*SD*)	96.72	(10.22)										
System maintenance *M* (*SD*)	57.77	(7.90)										
Mother report *N*	82											
Relationship *M* (*SD*)	98.05	(10.59)										
System maintenance *M* (*SD*)	54.91	(7.25)										
Father report *N*	45											
Relationship *M* (*SD*)	98.27	(8.36)										
System maintenance *M* (*SD*)	54.78	(6.28)										

7
